# Teaching Emotional Intelligence: A Control Group Study of a Brief Educational Intervention for Emergency Medicine Residents

**DOI:** 10.5811/westjem.2015.8.27304

**Published:** 2015-11-22

**Authors:** Diane L. Gorgas, Sarah Greenberger, David P. Bahner, David P. Way

**Affiliations:** Ohio State University College of Medicine, Department of Emergency Medicine, Columbus, Ohio

## Abstract

**Introduction:**

Emotional Intelligence (EI) is defined as an ability to perceive another’s emotional state combined with an ability to modify one’s own. Physicians with this ability are at a distinct advantage, both in fostering teams and in making sound decisions. Studies have shown that higher physician EI’s are associated with lower incidence of burn-out, longer careers, more positive patient-physician interactions, increased empathy, and improved communication skills. We explored the potential for EI to be learned as a skill (as opposed to being an innate ability) through a brief educational intervention with emergency medicine (EM) residents.

**Methods:**

This study was conducted at a large urban EM residency program. Residents were randomized to either EI intervention or control groups. The intervention was a two-hour session focused on improving the skill of social perspective taking (SPT), a skill related to social awareness. Due to time limitations, we used a 10-item sample of the Hay 360 Emotional Competence Inventory to measure EI at three time points for the training group: before (pre) and after (post) training, and at six-months post training (follow up); and at two time points for the control group: pre- and follow up. The preliminary analysis was a four-way analysis of variance with one repeated measure: Group x Gender x Program Year over Time. We also completed post-hoc tests.

**Results:**

Thirty-three EM residents participated in the study (33 of 36, 92%), 19 in the EI intervention group and 14 in the control group. We found a significant interaction effect between Group and Time (p≤0.05). Post-hoc tests revealed a significant increase in EI scores from Time 1 to 3 for the EI intervention group (62.6% to 74.2%), but no statistical change was observed for the controls (66.8% to 66.1%, p=0.77). We observed no main effects involving gender or level of training.

**Conclusion:**

Our brief EI training showed a delayed but statistically significant positive impact on EM residents six months after the intervention involving SPT. One possible explanation for this finding is that residents required time to process and apply the EI skills training in order for us to detect measurable change. More rigorous measurement will be needed in future studies to aid in the interpretation of our findings.

## INTRODUCTION

Emotional intelligence (EI) is a complex construct involving the perception, processing, regulation and management of emotions.^1^ Mayer and Salovey define EI “as a four dimensional construct, comprising the abilities to perceive emotion, use emotion to facilitate thought, understand emotions, and manage emotion.”^2^ EI is a construct distinctly different from personality or general intelligence, and has been shown to be a practical predictor of job performance within the most competitive professions such as business, engineering, or medicine.^3^–^5^

EI is thought to involve four distinct measurable skill sets: self-awareness, self-management, social awareness, and relationship management.^9^ While some debate exists as to whether EI is a state (learned) or trait (innate), we conceive of EI as a fluid construct, susceptible to improvement or decline.^7^–^10^

Although EI is a distinctly different construct than cognitive intelligence (sometimes expressed as intelligence quotient (IQ), and derived from a score on a mental abilities test), the two are thought to have a complex interaction when involved with decision-making in an emotionally charged environment.^1^–^3^ In an emotionally charged environment, individuals with both high EI and IQ are thought to be able to mediate the effects of their own, and others’ emotions in order to make sound judgments.^1^

### EI and Physician Development

For physicians, EI’s pattern of change over time runs counter to change for individuals in other fields.^11^–^14^ Some studies have demonstrated that physician EI deteriorates over time and as training progresses, while professionals in other fields tend towards improvements in EI over time and with experience.^12^,^14^,^15^ Deterioration in physician EI has been attributed to de-sensitization effects from medical training, de-personalization of the patient-physician relationship, and burnout.^1^,^11^,^12^,^15^ Burnout, which can lead to premature career termination, is a common problem in medicine with reported rates as high as 50% in medical students, 75% for residents, and 60% in practicing physicians.^11^,^15^

A growing body of literature examines the relationship of EI to resident physician wellness, how it relates to resilience during training, and how it impacts career success including career satisfaction and career longevity.^11^,^12^,^14^,^15^ EI in and of itself has been shown to be a predictor of success in residency training.^11^ Residents with low EI are ill-equipped to cope with the stressors of training and practice, and tend to leave the profession earlier.^5^,^11^,^15^,^16^ Residents with high EI have been shown to have more pro-social behavior, better academic performance, better relationships with patients, and longer careers.^11^,^15^

### Strategies for Teaching EI

Teaching strategies to improve an individual’s EI vary from simple training in identifying nonverbal cues for emotions, to more advanced exercises in self-reflection and identification of situational stressors.^9^,^10^,^17^,^18^ Workplace interventions include education about burnout, workload modifications, increasing the diversity of work duties, stress management training, mentoring, team-building, communication skills, and wellness workshops.^19^–^21^ The ideal intervention for professionals would effectively improve EI in the workplace without huge investments of time and resources.

Successful EI training programs have been conducted at both the undergraduate and graduate medical education levels, suggesting that interventions can be effective during these periods of physician development.^17^,^20^–^23^

Simulated patient encounters with feedback have been used to teach individuals, whereas small-group discussion formats have been used to develop EI collectively across groups.^20^,^22^–^24^ Some studies have shown that female students are more amenable to and gain more from EI training than their male counterparts.^23^ Some EI training has yielded mixed or ineffective results, including some with considerable expenditures of effort and time.^16^ Generally, the most effective training has been coupled with clinical experience.^17^,^22^

### Measuring EI

There are numerous instruments and scales that purport to measure EI or components of EI. MacCann et. al. reviewed four primary EI instruments including the Emotional Quotient Inventory (EQ-i), the Schutte Self-Report Inventory (SSRI), the Hay 360 Emotional Competence Inventory (ECI), and the Mayer, Salovey, Caruso EI Test (MSCEIT).^25^ The instruments are based on slightly varied conceptualizations of the EI construct. The MSCEIT treats EI as a trait or ability, while the other three are considered mixed models because some of the items resemble those found on conventional personality tests, while others are more related to skills or competencies.^25^ The instruments vary widely on the composition of their parts or subscales, response options, tasks, stimuli, and methods for scoring.^25^ All four EI assessment instruments are comprehensive, complex, and time-consuming.^25^

### Purpose

The purpose of this study was to measure the effects of a brief educational intervention designed to improve the general EI of EM residents. Specifically, we taught a group of residents one EI-related skill called social perspective-taking to see if it would improve their EI as operationalized by scores on a 10-item sample from the Hay 360 ECI.^26^,^27^

## METHODS

This study was conducted at a large, academic, urban EM residency training program in the Midwest, during academic year 2011–12. The residency is a three-year program, with 12 residents per class, post-graduate year (PGY) 1–3. Residents at all three levels participated in the study, which was reviewed and approved by our institution’s behavioral sciences institutional review board.

### Study Design

We used a pre-test, post-test, six-month follow-up randomized control group design. EM residents were assigned to either an intervention (experimental) group or a control group. The assignments were random, but stratified to assure that both groups had equal numbers of residents from each class and each gender. Both groups were given the sample Hay 360 ECI as a pre-test. The intervention group completed the EI training session and completed the sample Hay 360 ECI as a post-test. Both the intervention and control groups completed the sample Hay 360 ECI as a six-month follow-up assessment.

### Educational Intervention

As are most EM residency curricula, ours is densely packed with compulsory content material. Subsequently, our EI intervention and assessments needed to fit into two conference sessions of two hours in length, offered six months apart and alternated with another program activity. Accordingly, our intervention group participated in the EI training in the first two-hour session, and the control group received the training six months later in the second two-hour session.

Time restrictions required us to limit instruction to one sub-skill from one of the primary EI skills, social awareness. We focused on social awareness because of its importance in the clinical setting, and because of its relationship to communication. Social awareness encompasses the sub-skills of empathy, organizational awareness, and social perspective taking (SPT). SPT is a skill related to understanding another individual’s viewpoint. Someone with skill in SPT can “accurately infer the thoughts and feelings of others.”^4^,^5^,^18^,^28^,^29^ SPT has been shown to predict better communication skills in practitioners and trainees in both nonmedical and medical careers.^5^,^28^–^30^ We hypothesized that the associated sub-skill of SPT could be taught within our available time limits and that focus on this particular skill would lead to measureable results.

We would have preferred to use the Hay 360 ECI as our measure of EI for three primary reasons. First, the conceptual framework of the instrument was most consistent with our conceptual framework of EI. Second, the assessment uses scenarios as prompts and partial credit scoring, which fit with our repeated measures research design. In other words, we felt that this format would be the least likely to yield a practice effect. Third, the ECI included subscales most directly related to our instruction: empathy, social awareness and SPT.

However, due to cost in time and money, we were restricted to using a measure that was brief, and freely available. Fortunately, we were able to identify a short, sample version of the Hay 360 ECI called The Hay 360 EI Quiz. This was available on the publisher’s website, and it took less than 10 minutes for the residents to complete (see Appendix A).^31^

At the beginning of the first EI intervention session, both groups completed the 10-item sample Hay 360 ECI assessment. The control group was then dismissed to a separate conference room for an unrelated residency program activity, while the treatment group remained for the EI intervention.

The EI intervention was modeled after similar programs for physicians and trainees in internal medicine and family medicine.^15^,^20^ Due to time limitations, we chose to focus our training on two very specific components of EI, compassion and SPT.^4^,^28^,^32^ The session was introduced with a lecture covering basic EI vocabulary and concepts, a description of environmental stressors common to EM residents, and then a brief description about the intended benefits of EI training activities. A video of an interview with Daniel Goleman a leading authority on the topic of EI, and a video of a TED-Talk lecture delivered by Daniel Goleman on the topic of compassion were interspersed into the introductory lecture.^5^,^32^ The lecture was followed by a series of four case scenarios, each involving a person in distress. The cases included a list of suggested actions that an external observer might take, including the reasons why they might take that action.

The first two cases were presented and discussed with the entire intervention group. Participants were asked questions about the cases to guide them to identify the source of distress, the cause of the distress, potential environmental factors, and how the perspective one takes might impact their response to the situation. Two subsequent cases were discussed in facilitated small groups. At the end of the session, each small group presented their case analysis, their response, and a defense of their response. The session concluded with a debriefing in which the “best” responses to each case were identified along with explanations. (See Appendix B for more thorough description of the program.)

The 10-item sample Hay 360 ECI survey was administered to the intervention group at the conclusion of the intervention session.^31^ Over the following six months, during their regular clinical work in the emergency department, residents in both groups received regular clinical performance feedback including that which involved ACGME Milestones in interpersonal communication. Both the intervention group and control group retook the 10-item sample Hay 360 ECI survey at the end of the six-month period.

### Data Analysis

The overall study design involved four independent variables: group (intervention vs. control); gender (female vs. male); program year (1 vs. 2 and 3); and time (1: pre-intervention, 2: post-intervention, and 3: six-month follow up). There was one dependent variable, the sample Hay 360 ECI as a measure of EI. The preliminary omnibus analysis was a 4-way analysis of variance (ANOVA) with one repeated factor (time). Since both groups (intervention and controls) were not measured at all three time periods, we dropped time 2 from this analysis.

Due to the repeated measure nature of our design, we used partial eta^2^ for computations of effect size, as suggested by Brown.^33^ We further analyzed the significant two-way interaction of group x time with an analysis of the simple effects of groups separately using paired t-tests.^34^ Since the simple effect was significant for the intervention group only, we conducted a 1-way repeated measures ANOVA as a post-hoc analysis of their sample Hay 360 ECI score change over time.

## RESULTS

Thirty-three EM residents participated in the study (33 of 36, 92%), 19 in the EI intervention group and 14 in the control group. Participants were evenly split by gender (16 women, 17 men), and by program year (13 PGY-1, 9 PGY-2, and 11 PGY-3) (Table 1). Due to scheduling conflicts, we ended up with slightly more PGY-3 residents in the intervention group. So to verify the equivalence of the groups at baseline, we ran an independent t-test and found the two groups to be statistically equivalent, (Intervention Mean=62.6; Control Mean=66.8; t= −1.02; df=31; p=0.316).

**Figure 1:**
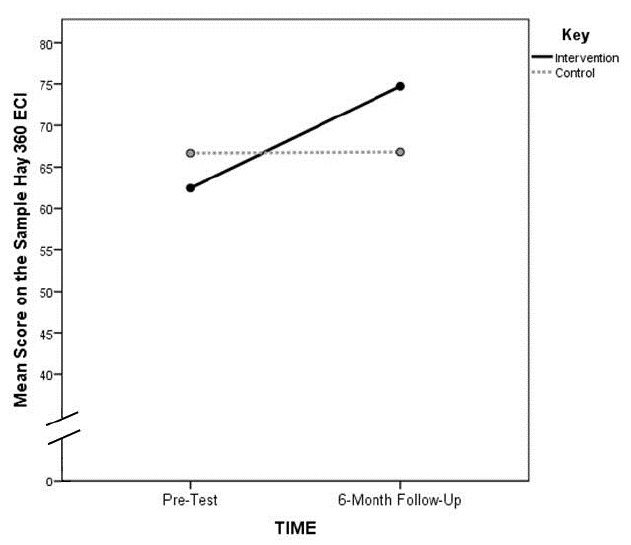
Line graph of the 2-Way Interaction between groups of Emergency Medicine Residents (intervention vs. control) and time (EI pre-test and EI six-month follow up). *ECI,* emotional competence inventory

The four-way ANOVA with one repeated measure resulted in a significant interaction effect between group and time (F=7.16_(1,21)_; p≤0.05). No other main effects or interactions were statistically significant (Tables 2, 3a, and 3b). The means plot (Figure 1) of this interaction shows a marked increase in EI score for the intervention group and no detectable change in EI score for the control group. The partial eta^2^ effect size of the group x time interaction was 0.25, which is considered large.^35^

We analyzed the significant interaction with an analysis of simple effects. This was accomplished through paired t-tests of each group’s pre- and post-EI score for each group separately. We found that the change in EI mean scores for the intervention group from Time 1 to Time 3 (62.6 to 74.2) was significant (t_1,18_= −3.54; p≤0.01) while the change over the same time period for the control group (66.8 to 66.1) was not (t_1,13_=0.31; p=0.77).

**Figure 2:**
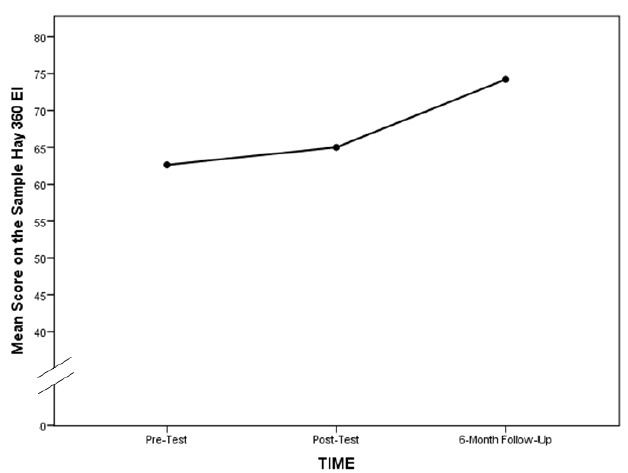
Line graph of the emergency medicine resident intervention group’s mean emotional intelligence scores over three time periods: pre-test, post-test, and six-month follow up. *EI,* emotional intelligence

Finally, we looked at just the intervention group’s mean scores over time with a one-way repeated measures ANOVA. The results showed that there was no significant change from Time 1 to Time 2 (Mean_1_=62.6; Mean_2_=65.0; F_1,18_=0.98; p=0.34), but there was a significant change from Time 2 to Time 3 (Mean_2_=65.0; Mean_3_=74.2; F_1,18_=7.81; p≤0.05) (see Figure 2). The associated partial eta^2^ effect size for this analysis was also large, 0.31.

To summarize, the significant interaction between group and time can be explained by a significant increase in the intervention group’s score on the 10-item sample of the Hay 360 ECI Test over the time before the intervention to the six-month follow up. The control group’s scores remained about the same over that period of time. The significant increase in EI for the treatment group was not detected immediately following the intervention; instead, it was observed six months after the EI training.

## DISCUSSION

We developed an educational intervention designed to improve EI as characterized by the skill of SPT. The intervention was customized for EM residents, and was intended to be brief, so as not to compete for curricular time with other topics. Our review of the literature yielded very few studies on attempts to improve EI in the EM resident population, a medical specialty that commonly works in an emotionally charged environment and is prone to professional burnout.^36^,^37^ Our hope is that this study can serve as impetus for continued efforts to study EI improvement in EM residents.

As expected, pre- and post-EI scores for our control group remained relatively stable. The stability in the control group’s scores over time, taken together with the changes in the intervention group’s scores indicate that the test was neither intuitive nor learned, so that our results cannot be attributed solely to testing effects. Additionally, the six-month period between our initial and final assessments represented a satisfactory washout period.

We did not detect significant improvement in EI scores immediately following the brief intervention. However, at testing six months post-intervention, the intervention group’s EI scores significantly improved, whereas the control group’s scores had not. Intuitively, we would have expected the training effect to be apparent at the initial post-intervention assessment. Instead, consistent with other studies, we found that residents required time to process and apply the EI skills training in the clinical setting.^17^,^22^ Perhaps the traditional feedback related to interpersonal communications and received through clinical performance evaluations was more meaningful to the treatment group when compared to the control group.

Prior literature has demonstrated that in the general population, EI improves over a lifetime and is influenced by life experience.^4^,^11^–^14^ However, in physicians, research has shown that EI tends to decrease over time and particularly during the course of residency training.^11^–^14^ As such, the gains in physician EI we observed after our intervention represent a notable reversal of the decline in EI normally associated with physician development through residency training.

## LIMITATIONS

While we tried to study the impact of this brief intervention using an experimental design, there are certainly limitations to this study. First, we believe we did not observe significant main effects across gender or length of training because of the relatively small size of these subgroups within our population. However, we believe that collectively we had sufficient power to accurately assess the effects of the intervention over time. Further studies of the intervention’s effect on EM residents at other program sites and to residents in other specialties are needed to determine the generalizability of our findings.

Second, due to time limitations we designed our intervention to specifically focus on one small aspect of EI, the skill of SPT because of its relationship to engagement with others; i.e. you have to notice or observe distress in others in order to be able to act or make a compassionate choice.^32^ Yet our small intervention on only one sub-skill of the many that comprise EI was found to improve a score reflective of total EI. Further research is needed to help explain the nature of the relationship between SPT and total EI.

Finally, we acknowledge that a major limitation of this study is that we used a measurement instrument that was only a sample of the full battery of the Hay 360 ECI (or the ECI-U for university students). Further research is needed to determine whether the full battery ECI-U would have yielded the same results.

## CONCLUSION

In summary, we were able to observe improved scores on a measure of EI in EM residents with a brief intervention designed to improve the residents’ skill in SPT. Interestingly however, the improvement was not immediate and only observed after a six-month delay. We conclude from this finding that the SPT skill required additional practice with specific feedback about interpersonal communications in the clinical setting to be fully realized by our residents. Those who received the EI training were better able to internalize feedback on social interactions than were trainees without the brief intervention. Given the importance of physician communication skills to patient satisfaction, this research is all the more timely.

## Supplementary Information





## Figures and Tables

**Table 1:** Counts and percentages of 33 emergency medicine resident participants in emotional intelligence training by gender and program year.

	Control	Intervention	Total
Gender			
Female	8 (24.2)	8 (24.2)	16 (48.5)
Male	6 (18.2)	11 (33.3)	17 (51.5)
Total	14 (42.4)	19 (57.6)	33 (100)
Program year			
1	6 (18.2)	6 (18.2)	12 (36.4)
2	4 (12.1)	5 (15.2)	9 (27.3)
3	4 (12.1)	8 (24.2)	12 (36.4)
Total	14 (42.4)	19 (57.6)	33 (100)

**Table 2:** Analysis of variance (ANOVA) results of emotional intelligence measures of 33 emergency medicine residents comparing intervention group, gender and program year level (PGY) at two time periods: Before Intervention, and six-months later.

Source: 4-Way ANOVA with 1 repeated factor (Time)	F	P value	Effect size*
Main effects			
Group (experimental vs. control)	0.26	NS	
Gender	1.01	NS	
PGY level	1.02	NS	
Time (pre-intervention vs. 6-month follow-up)	7.49	0.012	0.263
2-way interactions			
Gender x time	0.03	NS	
Group x time	7.16	0.014	0.254
PGY level x time	0.33	NS	
Group x gender	1.25	NS	
Group x PGY level	0.13	NS	
Gender x PGY level	0.88	NS	
3-way interactions			
Gender x group x time	0.15	NS	
PGY level x group x time	1.03	NS	
PGY level x gender x time	1.77	NS	
Group x PGY level x gender	0.18	NS	
4-way interactions			
Gender x group x PGY level x time	0.94	NS	

*NS*, not significant

* Partial Eta Squared

**Table 3a:** Pre emotional intelligence score. Descriptive statistics (mean, standard deviation [SD], and number) of emotional intelligence measure for 33 emergency medicine residents broken down by gender, post-graduate year (PGY) and intervention/control groups before intervention.

Group	PGY-level	Mean	SD	N
Female				
Intervention	PGY 1	55.0	13.2	3
	PGY 2	70.0	14.1	2
	PGY 3	50.0	17.3	3
	Total	56.9	15.3	8
Control	PGY 1	65.0	18.0	3
	PGY 2	72.5	10.6	2
	PGY 3	65.0	5.0	3
	Total	66.9	11.3	8
Total	PGY 1	60.0	15.2	6
	PGY 2	71.3	10.3	4
	PGY 3	57.5	14.1	6
	Total	61.9	14.0	16
Male				
Intervention	PGY 1	68.3	2.9	3
	PGY 2	63.3	5.8	3
	PGY 3	68.0	7.6	5
	Total	66.8	6.0	11
Control	PGY 1	70.0	17.3	3
	PGY 2	62.5	10.6	2
	PGY 3	65.0	0.0	1
	Total	66.7	12.5	6
Total	PGY 1	69.2	11.1	6
	PGY 2	63.0	6.7	5
	PGY 3	67.5	6.9	6
	Total	66.8	8.5	17
Total				
Intervention	PGY 1	61.7	11.3	6
	PGY 2	66.0	8.9	5
	PGY 3	61.3	14.3	8
	Total	62.6	11.7	19
Control	PGY 1	67.5	16.0	6
	PGY 2	67.5	10.4	4
	PGY 3	65.0	4.1	4
	Total	66.8	11.4	14
Total	PGY 1	64.6	13.6	12
	PGY 2	66.7	9.0	9
	PGY 3	62.5	11.8	12
	Total	64.4	11.6	33

**Table 3b:** Six-month follow-up emotional intelligence score. Descriptive statistics (mean, standard deviation [SD], and number) of EI measure for 33 emergency medicine residents broken down by gender, post-graduate year (PGY) and intervention/control groups six months after intervention.

Group	PGY-level	Mean	SD	N
Female				
Intervention	PGY 1	66.7	5.8	3
	PGY 2	75.0	14.1	2
	PGY 3	71.7	7.6	3
	Total	70.6	8.2	8
Control	PGY 1	61.7	14.4	3
	PGY 2	72.5	3.5	2
	PGY 3	65.0	5.0	3
	Total	65.6	9.4	8
Total	PGY 1	64.2	10.2	6
	PGY 2	73.8	8.5	4
	PGY 3	68.3	6.8	6
	Total	68.1	8.9	16
Male				
Intervention	PGY 1	85.0	5.0	3
	PGY 2	80.0	5.0	3
	PGY 3	70.0	11.7	5
	Total	76.8	10.6	11
Control	PGY 1	61.7	22.5	3
	PGY 2	75.0	0.0	2
	PGY 3	65.0	0.0	1
	Total	66.7	15.7	6
Total	PGY 1	73.3	19.4	6
	PGY 2	78.0	4.5	5
	PGY 3	69.2	10.7	6
	Total	73.2	13.1	17
Total				
Intervention	PGY 1	75.8	11.1	6
	PGY 2	78.0	8.4	5
	PGY 3	70.6	9.8	8
	Total	74.2	9.9	19
Control	PGY 1	61.7	16.9	6
	PGY 2	73.8	2.5	4
	PGY 3	65.0	4.1	4
	Total	66.1	12.0	14
Total	PGY 1	68.8	15.5	12
	PGY 2	76.1	6.5	9
	PGY 3	68.8	8.6	12
	Total	70.8	11.4	33
