# Supplementary material for: Teaching Emotional Intelligence: A Control Group Study of a Brief Educational Intervention for Emergency Medicine Residents
Source: West J Emerg Med. 2015 Nov 22;16(6):899–906. doi: 10.5811/westjem.2015.8.27304 (PMC4651591; doi:10.5811/westjem.2015.8.27304)
Supplement: Supplementary file 1 [file wjem-16-899-s001.pdf]

Hay Group Transforming Learning EI Quiz
https://atrium.haygroup.com/us/quizzes/emotional-intelligence-quiz.aspx

Emotional Intelligence

Emotional and social intelligence makes the difference between a highly effective leader and an average one. It helps managers and professionals create competitive advantage for their organizations by increasing performance, innovation and teamwork, ensuring time and resources are used effectively, and building motivation and trust.

Directions: Answer the following questions to get a glimpse into your own experiences at work and their impact on your effectiveness.

1. You are on an airplane that suddenly hits extremely bad turbulence and begins rocking from side to side. What do you do?

   a) Continue to read your book or magazine, or watch the movie, trying to pay little attention to the turbulence. [10]

   b) Become vigilant for an emergency, carefully monitoring the stewardesses and reading the emergency instructions card. [10]

   c) A little of both a and b. [10]

   d) Not sure - never noticed. [0]

2. You are in a meeting when a colleague takes credit for work that you have done. What do you do?

   a) Immediately and publicly confront the colleague over the ownership of your work. [0]

   b) After the meeting, take the colleague aside and tell her that you would appreciate in the future that she credits you when speaking about your work. [5]

   c) Nothing, it's not a good idea to embarrass colleagues in public. [0]

   d) After the colleague speaks, publicly thank her for referencing your work and give the group more specific detail about what you were trying to accomplish. [10]

3. You are a customer service representative and have just gotten an extremely angry client on the phone. What do you do?

   a) Hang-up. It doesn't pay to take abuse from anyone. [0]

   b) Listen to the client and rephrase what you gather he is feeling. [5]

   c) Explain to the client that he is being unfair, that you are only trying to do your job, and you would appreciate it if he wouldn't get in the way of this. [0]

   d) Tell the client you understand how frustrating this must be for him, and offer a specific thing you can do to help him get his problem resolved. [10]

4. You are a college student who had hoped to get an A in a course that was important for your future career aspirations. You have just found out you got a C- on the midterm. What do you do?

   a) Sketch out a specific plan for ways to improve your grade and resolve to follow through. [10]

   b) Decide you do not have what it takes to make it in that career. [0]

   c) Tell yourself it really doesn't matter how much you do in the course, concentrate instead on other classes where your grades are higher. [5]

   d) Go see the professor and try to talk her into giving you a better grade. [0]

5. You are a manager in an organization that is trying to encourage respect for racial and ethnic diversity. You overhear someone telling a racist joke. What do you do?

   a) Ignore it - the best way to deal with these things is not to react. [0]

   b) Call the person into your office and explain that their behavior is inappropriate and is grounds for disciplinary action if repeated. [5]

   c) Speak up on the spot, saying that such jokes are inappropriate and will not be tolerated in your organization. [10]

   d) Suggest to the person telling the joke he go through a diversity training program. [5]

6. You are an insurance salesman calling on prospective clients. You have left the last 15 clients empty-handed. What do you do?

   a) Call it a day and go home early to miss rush-hour traffic. [0]

   b) Try something new in the next call, and keep plugging away. [10]

   c) List your strengths and weaknesses to identify what may be undermining your ability to sell. [5]

   d) Sharpen up your resume. [0]

7. You are trying to calm down a colleague who has worked herself into a fury because the driver of another car has cut dangerously close in front of her. What do you do?

   a) Tell her to forget about it-she's OK now and it is no big deal. [0]

   b) Put on one of her favorite tapes and try to distract her. [0]

   c) Join her in criticizing the other driver. [5]

   d) Tell her about a time something like this happened to you, and how angry you felt, until you saw the other driver was on the way to the hospital. [10]

8. A discussion between you and your partner has escalated into a shouting match. You are both upset and in the heat of the argument, start making personal attacks which neither of you really mean. What is the best thing to do?

   a) Agree to take a 20-minute break before continuing the discussion. [10]

   b) Go silent, regardless of what your partner says. [0]

   c) Say you are sorry, and ask your partner to apologize too. [0]

   d) Stop for a moment, collect your thoughts, then restate your side of the case as precisely as possible. [0]

9. You have been given the task of managing a team that has been unable to come up with a creative solution to a work problem. What is the first thing that you do?

   a) Draw up an agenda, call a meeting and allot a specific period of time to discuss each item. [0]

   b) Organize an off-site meeting aimed specifically at encouraging the team to get to know each other better. [10]

   c) Begin by asking each person individually for ideas about how to solve the problem. [0]

   d) Start out with a brainstorming session, encouraging each person to say whatever comes to mind, no matter how wild. [5]

10. You have recently been assigned a young manager in your team, and have noticed that he appears to be unable to make the simplest of decisions without seeking advice from you. What do you do?

   a) Accept that he "does not have what it takes to succeed around here" and find others in your team to take on his tasks. [0]

   b) Get an HR manager to talk to him about where he sees his future in the organization. [5]

   c) Purposely give him lots of complex decisions to make so that he will become more confident in the role. [0]

   d) Engineer an ongoing series of challenging but manageable experiences for him, and make yourself available to act as his mentor. [10]

* Please note: this quiz is designed to be a quick introduction to the importance of emotional intelligence (EI). The results you get from this quiz are not a comprehensive picture of your EI and the quiz is not representative of Hay Group surveys.
