# Supplementary material for: Teaching Emotional Intelligence: A Control Group Study of a Brief Educational Intervention for Emergency Medicine Residents
Source: West J Emerg Med. 2015 Nov 22;16(6):899–906. doi: 10.5811/westjem.2015.8.27304 (PMC4651591; doi:10.5811/westjem.2015.8.27304)
Supplement: Supplementary file 2 [file wjem-16-899-s002.pdf]

Appendix B.

Emotional Intelligence Session

Overview & Objectives

- Be able to define Emotional Intelligence (EI) and explain its relationship between IQ.
- Be able to explain the difference between a trait (ability) and state (skill) construct.
- Be able to list the components of EI and their manifestations in the workplace (self-awareness, self-management, social awareness, relationship management)
- Be able to explain the impact of EI on patient satisfaction, job satisfaction, problem solving, and career longevity
- Be able to apply the EI vocabulary and conceptual constructs to each of the case vignettes that feature an EI concept.
- Be able to explain how SPT contributes to selecting an action in each of the case vignettes.

Faculty Staffing

- 3 faculty presenters and small group facilitators (3-4 faculty rotating between groups)

Materials

- Goleman videos 1) Emotional Intelligence and 2) Compassion
- Hay Group 360 EI Quiz
- Consent forms
- Powerpoint slides
- Faculty discussion leader guide

Methods (Schedule)

I. Introduction (5 minutes)

II. What is Emotional Intelligence? (Brief Lecture) (15 minutes)

III. View Goleman video on EI definition (5minutes)

IV. Large Group Case Discussions (20 minutes)
- Discussion of cases 1 and 2

V. Small Group Case Discussions (20 minutes)
- Discuss cases 3 and 4 in facilitated small groups with focus on provided questions
- Small group summary presentations
    - State action you would take and reasons for that action

VI. Barriers to Compassion (Brief Introductory Lecture) (15 minutes)

VII. View Goleman Youtube Video on Compassion (15 minutes)

VIII. Barriers to Compassion Specific to **Emergency Medicine** (10 minutes)

IX. Wrap up (15 minutes)
- Complete Post-EI Assessment

Case Example

You come home to find your neighbor's 8-year-old daughter sitting on the steps of her front porch, sobbing loudly. The front door to her home is open and you can see your neighbor's car in the driveway. Do you:

a) Pretend you don't notice her, to avoid embarrassing her or meddling in her affairs.
b) Call your neighbor to discreetly make sure he is aware of his child's distress.
c) Ask the girl directly if she needs help or wants to talk about why she is upset.
d) Walk up to the front door, ring the doorbell, and speak with your neighbor in the girl's presence.

Guiding Questions for Facilitators:

1. What do you think the little girl is feeling? If her parents can hear her, what do you think they are feeling?

2. What questions do you wish you could have answered before choosing an action?

3. When should you get involved?

4. Would the situation be different if you are on the way out the door and late to work?

5. Does the situation depend on your relationship with your neighbor?

6. What role does the family's right to privacy play into your decision making process?

7. Does your responsibility differ from other neighbor's responsibility because you are a physician?

8. Would your response change if there was a history of domestic violence in the neighbor's household?
